# Generating ‘Omic Knowledge’: The Role of Informatics in High Content Screening

**DOI:** 10.2174/138620709789383259

**Published:** 2009-11

**Authors:** Mark A Collins

**Affiliations:** Cellular Imaging & Analysis, Thermo Fisher Scientific, USA

**Keywords:** High content screening, HCS, high content analysis, HCA, genomics, informatics, bioinformatics, ontology, systems biology, drug discovery.

## Abstract

High Content Screening (HCS) and High Content Analysis (HCA) have emerged over the past 10 years as a powerful technology for both drug discovery and systems biology. Founded on the automated**,** quantitative image analysis of fluorescently labeled cells or engineered cell lines**, **HCS provides unparalleled levels of multi-parameter data on cellular events and is being widely adopted**,** with great benefits**,** in many aspects of life science from gaining a better understanding of disease processes**,** through better models of toxicity**,** to generating systems views of cellular processes. This paper looks at the role of informatics and bioinformatics in both enabling and driving HCS to further our understanding of both the genome and the cellome and looks into the future to see where such deep knowledge could take us.

## INTRODUCTION

Completed in 2003, the Human Genome Project (HGP) resulted in the sequencing of the 30,000 genes contained in the entire human genome [1], while this was a remarkable effort, we are only at the beginning of our understanding of the role of all these genes in living systems. Functional genomics, the role of genes in complex traits and disease, gene regulation and complex systems biology are just some of the questions that were raised by the HGP and are the subject of much research. The cell can be considered as the simplest unit of life that is amenable to the study of many of the questions raised by the HGP. However “sequencing” the cell, i.e. deep understanding of cellular processes, interactions, signaling, death etc., represents a technological challenge akin to sequencing genes- a challenge many have termed the ‘cellome’. The emergence of HCS with its ability to quantitatively measure what, where and when an event occurs in a cell offers just that richness of data from which many of the key questions about gene function may be answered. However, in the same manner that genomics solved the problem of high throughput data acquisition but then hit a bottleneck with respect to infrastructure and tools to manage and mine that data for knowledge, so HCS is now reaching the same status.

HCS systems typically scan a multi-well plate with cells or cellular components in each well, acquire multiple images of cells, and extract multiple features (or measurements) relevant to the biology, resulting in a large quantity of data and images. The amount of data and images generated from a single microtiter plate can range from hundreds of megabytes (MB) to multiple gigabytes (GB). Large numbers of plates are typically analyzed in screening operations and large scale system biology experiments, often resulting in billions of features and millions of images with a need for multiple terabytes (TB) of storage in a short period of time.

While the management of this kind of data is becoming commonplace, tools to generate ‘omic knowledge from billions of cellular measurements are less mature and we believe may hinder HCS from achieving its full potential of solving the cellome.

Our goal in this chapter is to provide a brief overview of informatics for managing HCS data, then to provide a series of examples of the use of HCS to solve key discovery problems and how informatics and bioinformatics are playing a role in this. Finally we look to the future to see how computational modeling and simulation could impact our insight of the cellome.

## THE MID MODEL FOR HCS INFORMATICS

In order to best describe the role of informatics and bioinformatics and the impact on driving the adoption and penetration of HCS into discovery, we have considered tiers of functionality, with each tier contributing to the overall systems view.

- **Management** – this tier provides the foundation for all the other tiers and deals with more of the informatics infrastructure for handling large data sets of considerable complexity together with associated images
- **Interpretation** – builds on the management tier and deals with primarily informatics tools, techniques and algorithms for analysis of multivariate HCS data to yield cell biology insights
- **Discovery** – the highest tier deals with bioinformatics and data mining tools to allow cellular knowledge generation in large scale multi-discipline studies.

This MID model provides a simple way to discuss the relative functionality needed to appropriately deal with HCS data and the impact derived in terms of gaining value from HCS.

## MANAGEMENT

HCS data, derived from some form of automated instrument can easily consume many terabytes of disk space. HCS data can be classified into 3 categories [2]

1. Image Data – these are the images acquired at each channel for each field within a well.
2. Derived Data – these are the measurements that result from performing an analysis on an image with image analysis algorithms (e.g., well features, cell features, etc.).
3. Meta-data – these are the associated data that provide context for the other two categories of data (i.e., meta-data is data that describes other data). For example, assay type, plate information, protocols, operators, calculated data such as dose response values, as well as annotations imported from other systems (e.g., sample identifiers and properties).

From a data volume perspective, the data to be saved per sample is primarily based on the Image data and the Derived data, Meta data is negligible in proportion from a storage perspective but is highly value for providing context.

Management of HCS data is the foundation of being able to derive value from it. Poor data management impacts both the ease of performing the other steps as well as the scientific robustness of the conclusions drawn from that data. Fig. (**1**) shows how HCS data must be considered together with other kinds of data in order for true value to be obtained. Ideal HCS data management ensures that all these disparate sources of data can be federated together to provide the knowledge with which to make biological decisions.

From an information technology perspective, management of large volumes of images and associated derived and meta-data represents a challenge and certainly when HCS was in its infancy this issue represented a bottleneck to the adoption of HCS. However, for the most part, with the move towards reliable, scalable ‘n’ tier architectures for HCS image and data management [2], this bottleneck has mostly been mitigated.

However, there remain two major challenges to HCS data management that will need to be solved if the cellome is to be achieved, the challenges relate to the format of HCS data storage, and how HCS data can be integrated in meaningful ways with other data such as chemical structures, gene sequences and pathway information. Key to solving both these issues is the role of standards. Many disciplines such as genomics and proteomics have proposed and adopted standards for recording and describing data, chief amongst these standards are the Minimum Information Standards [3-5] as well as the Open Microscopy Environment (OME) for describing data derived from images [6]. The minimum information and the OME standards provide a way to both store HCS data and images in an open and self describing format (XML) to facilitate data interchange such that images and meta-data acquired on one platform may be analyzed and interpreted by a wide variety of tools. Flow cytometry has used standard file formats and data models for many years [7] which has resulted in a variety of tools to analyze flow data. In many ways flow data is very comparable to HCS Derived and Meta data and so many of tools used for flow data may well contribute to the analysis of HCS data. Recently the flow community proposed its own minimum information standard (MIFlowCyt) [8].

The HCS community has yet to adopt a standard though MIAHA (Minimum Information about a High Content Assay) has been proposed [9].

Once data are available in an open and self describing form, analysis and interpretation are possible, however, key to this is the semantic meaning of the data. Whole cellome analysis of HCS data, perhaps bringing together data from different HCS platforms, imaging tools as well as data sources (chemical structures, gene sequences) will not be possible unless clear semantic meaning can be assigned. Indeed this is not just a challenge for HCS, genomics and proteomics face many of the same issues. Overcoming the problem of assigning meaning requires the use of ontologies, essentially frameworks of controlled vocabularies that provide annotations to data and meta data to facilitate analysis. The Open Biological Organization (OBO) [10] for example defines multiple ontologies for biology and brings together a number of previously developed ontologies (e.g., GO – the gene ontology). Once ontologies are agreed they can be adopted as part of the standards process.

## INTERPRETATION

To be clear we discuss here interpretation and analysis of the derived and meta data, there is no discussion of the image analysis involved in HCS.

Leveraging the foundation of robust data management for HCS, the analysis tier provides the basic insights into HCS data, allows for quality control and statistical analyses and most importantly utilizes the multi-parameter data to make decisions. It is also important to consider that, knowledge generation from HCS data must be considered a multistep workflow, requiring a number of functional stages from the basic gathering of data through QA, visualization, annotation and data mining. Fig. (**2**) explains these functional units and we discuss the tiers in the context of that workflow.

### Basic Interpretation of HCS Data

**Fig. (2):**
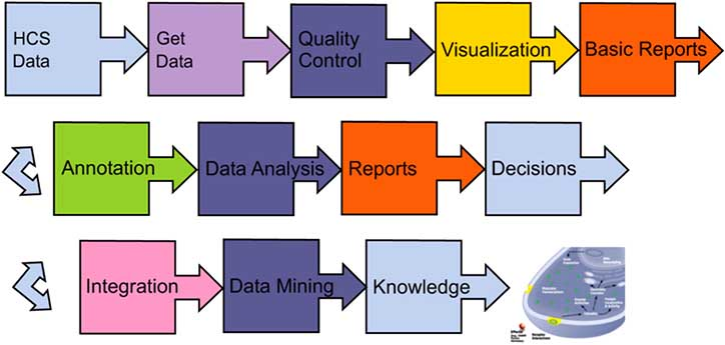
Workflow in HCS knowledge generation requires an integrated set of functional steps. Informatics and bioinformatics software must provide some or all of these functional steps in order for knowledge generation to be facilitated. The workflow may require a number of tools. Such integrated workflows benefit from standard approaches to data integration.

**Fig. (3):**
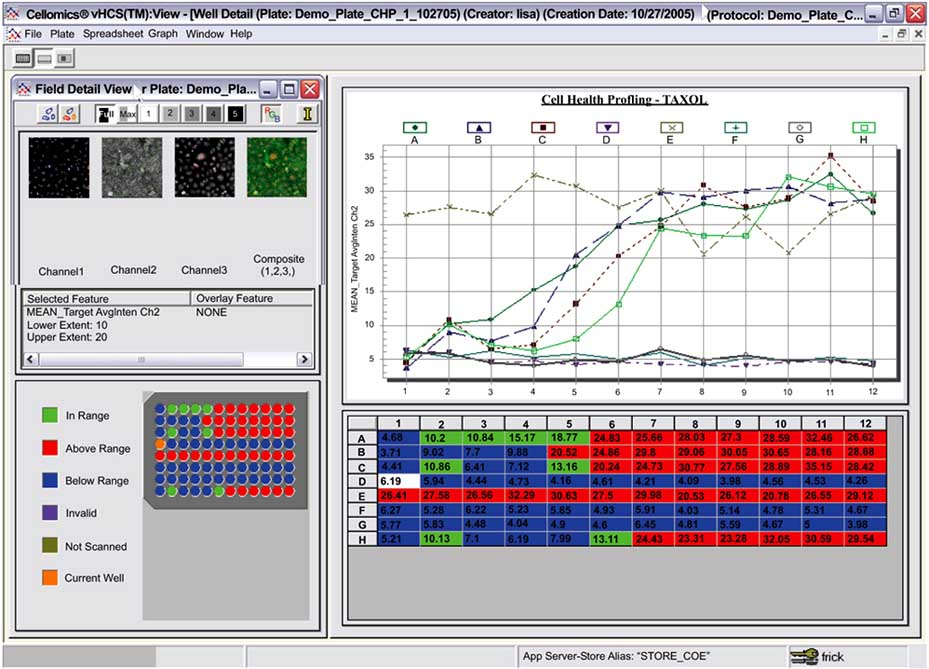
Multiple views on HCS data allow complex data sets to be easily visualized. (Fig. **3**) shows Thermo Scientific Cellomics® View analyzing well based data for a number of measurements of cell health. Use of graphics, color and a link to the image with masks (overlays) is shown here. Dose response data is also shown in the top right panel.

Basic interpretation of data covers the initial part of the HCS workflow from quality control, visualization and basic reporting to annotations (Fig. **2**). Such relatively simple functional steps have allowed HCS to be well integrated into many lead selection campaigns, either at the primary screening stage or at the secondary screening and lead prioritization stages. Although good tools exist for analysis of HTS data [12], some of which can be applied to HCS, HCS data presents some interesting challenges and opportunities, due to the more than one value per well. Early analyses of HCS data focused on reducing the data to a single measurement at the well level, and this reflected the fact that early on, HCS was often the only way to perform a hitherto intractable assay (e.g., neurite outgrowth). The multiple measurements of HCS allow not only activity at the target to be elucidated, but simple toxicity (measuring cell number for example) as well as off target effects to be determined. Traditional HTS approaches, such as thresholds based on descriptive statistics ignore the value of this multi parameter data, yet simple statistical and data visualization methods can be used to refine hit selection for HCS [11]. Allowing scientists to merely view all the HCS data in a variety of visualizations together with the image provides useful information on small numbers of plates and allows the user to drill down though the well level multi-parameter data to the cell subpopulation data (Fig. **3**). Use of data visualization tools such as Spotfire^®^ allow more sophisticated visualizations, Fig. (**4**) shows a simple viewing technique where the target measurement is represented in one color and the number of cells in the well is the size of the spot. More sophisticated 3D plots can also quickly visualize multiple parameters, Fig. (**5**), providing useful toxicity data in addition to the target measurements. In addition to filtering and visualization, the concept of building rule sets to analyze multiple parameters can also be employed. For example, it has been possible to successfully classify hits in toxicology into late stage, early stage and reversible status [14] by simply using Boolean rule sets (i.e. parameter 1 >=50 AND parameter 2 <30 OR parameter 3 >=200). Such rule sets are based on *a priori* knowledge however decision trees that are capable of being learned from data [13] offer greater flexibility and can often elucidate subtle effects.

The plethora of measurements possible with HCS using sophisticated image analysis often needs to be reduced to a smaller subset so as to determine the key parameters that separate the stimulated (positive biological effect) from the un-stimulated (control effect). It is commonplace to make a number of measurements of the stimulated/un-stimulated biology during assay development and then determine which are the top parameters that separate those states. In an internal study at Thermo Fisher Scientific, we employed T-tests, Z’ measurements, Self Organizing maps (SOM) and K-nearest neighbor (K-NN) analyses to determine the optimal set of morphological parameters. Fig. (**6**) shows the results of using K-NN to separate un-stimulated populations from stimulated populations. The K-NN identifies 3 key parameters (from a set of 52) that allow maximal separation. Such data reduction techniques can then be used to reduce the number of measurements made in a screening campaign without losing any discriminatory power, while maintaining manageable data set sizes in screens that may generate billions of data points.

Whole well analysis of multiple parameters, while more sophisticated than a single number, ignores the value of the subpopulation effects inherent in cell based imaging assays. While descriptive statistics such as mean, median, standard deviation and standard error provide some insight into the variation of the underlying cell data, more powerful statistics such as K-S (Kolmogorov-Smirnov) [15] have been widely adopted to compare the significance of distributions of cell populations for up to two parameters across experimental conditions, e.g., test *vs* control. While these techniques still reduce the data to a single number they provide increased confidence that the single number reflects the cell based data variation and the K-S statistic has been used successfully by a variety of studies [16-18].

In probably the first example of leveraging the power of more than one parameter in HCS studies. The authors [19] use relatively simple population density distributions of over 30 shape, texture and location measurements of cells against a range of concentrations of several known anticancer compounds. Plotting the natural log of these parameters for various concentrations of the drugs allowed a ‘high content profile’ to be generated that allowed easy comparison of drug effects on various cellular processes. Further visualizations such as quadrant plots, dot plots and scatter plots of cell based data revealed new insights into the interactions of drugs at the cell level in unprecedented detail. Similar visualizations of a number of cell measurements demonstrated that a panel of cell based assays could detect and classify threat agents based on cellular responses in those assays [20].

### The Power of Phenotypes

Early analyses of HCS data, described above, began to reveal the power of measuring multiple parameters and demonstrated that relatively simple statistics and visualizations (available in common informatics and statistical packages) could elegantly elucidate cellular responses.

It is now recognized that much of the power of HCS lies in generating cellular profiles or phenotypes from multivariate cell based data. Sophisticated informatics and bioinformatics techniques can be employed to analyses these phenotypes resulting in insights to cell biology. Such tools are represented further downstream in the HCS workflow (Fig. **2**) and build on the conclusions and insights made earlier in the workflow. Data quality control is of particular importance since data driven methods such as those detailed in this section require robust data sets to avoid poor performance and potentially misleading conclusions.

Classifiers of one type or another (e.g., supervised, unsupervised, statistical and machine learning) are very powerful techniques for analyzing multi-parameter data and have been successfully used for HCS. In a study of morphological effects of 107 compounds, known to inhibit protein kinases on a panel of 5 cell lines, Principal Component Analysis (PCA) of the morphological phenotypes following treatment with known kinase inhibitors identified a novel compound that inhibited CRB1, an enzyme involved in cell signaling. What was interesting was the fact that the phenotype detected was different from the cell phenotype of the known compound, yet the compounds differed chemically by only one hydroxyl group, indicating that HCS is able to clearly differentiate a minor structure difference on the basis of analysis of complex phenotypes [15]. Availability of such complex phenotypes and their analysis is key to realizing the potential of HCS data and utilizing the subtle effects of multiple cellular measurements. In another study [42], factor analysis of cell phenotypes based on cell cycle measures was used to profile a compound library and infer, based on the phenotypic profiles, mechanism of action of compounds. This work also demonstrates that phenotypic profiles are rich enough to provide biological meaning.

In addition to PCA, other techniques such as Hierarchical Clustering have been used to classify cellular phenotypes in response to both drug and RNAi treatments [21] furthering the impact of HCS in combinatorial biology experiments. Classifiers have also been shown to play a valuable role in predicting actives and non-actives in a screen. Several classifiers were trained on the cell profiles of known reference compounds and then the classifiers were used to predict actives and inactives in a screen for neurite outgrowth. A combination of K-nearest neighbors (K-NN), Fisher Linear Discriminant Analysis (LDA) and support vector machines were used to create a system able to predict an “active phenotype” in screens five times better than using traditional hit selection methods [22].

Highly complex data mining tools such as Support Vector Machines (SVM) [24] can be employed to analyze HCS data and may hold promise as they are tolerant to noise in data sets, a consideration of some importance for cell based measurements. SVMs have been successfully used to recognize phases of the cell cycle by classifying a set of fifty nine morphological measurements of cells. The SVM classification was compared with human annotations and demonstrated a high degree of accuracy and specificity in predicting mitotic sub-phases [23]. SVMs have also been successful in cellular multi-phenotypic mitotic analysis [39] as well as determining the best segementation of images from morphological measurements.

There is no doubt that classification of cellular phenotypes can begin to unlock the types of cellular knowledge that are useful for both drug screening as well as systems biology [26].

### Discovering Knowledge About the Cellome

From a pure systems engineering standpoint, biology from the ecosystem level to the genome level is a highly interconnected network. Attempting to probe such a highly interconnected system using a reductionist approach ignores this richness of connections and limits our ability to generate valuable knowledge. HCS provides sophisticated multi-parametric probes that when coupled with powerful bioinformatics tools can yield an understanding of these connections.

Pathways represent some of the more complex connection networks and ones that are heavily involved in cell regulation. A key regulatory network is the cell cycle and using a combination of HCS measurements of morphological changes in cell and RNAi knockdown of genes involved in cell cycle regulation complex phenotypic data sets have been generated. Analysis of these data sets using a combination of clustering and functional annotations showed a number of new pathways and processes involved in cell cycle and cell-size regulation [27], and identified a new translational inhibitor of the Cyclin/Cdk pathway. Generating these kinds of insights utilized data not just from HCS but from FACS as well as gene annotation, functional assignments and so on. Such system wide analysis using sophisticated tools are beginning to be used with great benefit, in areas such as transcriptional changes in breast cancer cells [28], modeling Parkinson’s disease [29] as well as the search for therapies for hepatocarcinomas [30].

In recent years, pathway analysis and modeling tools have been adopted for a wide variety of approaches from elucidating pathways to genome wide association studies. For the most part these tools have used genomic data (expression profiles) as a data source [31], but there is an increasing demand to use HCS data as a model input.

In genome wide RNA screening of human kinases involved in neurodegeneration, HCS was used to identify candidate kinases involved in neurite outgrowth and retraction. The candidate kinases were then grouped and linked using pathway analysis software (PathwayArchitect – Strategene) to create a regulatory network of the kinases involved in signaling. By combining HCS data, RNAi knockdown and pathway analysis the authors were able to get the first overall picture of the signaling that occurs during neurite degeneration as well as identify novel cross-talk between unrelated signaling pathways. This would not have been possible without such sophisticated bioinformatics tools [32], and it is now clear that the richness of data available from HCS is starting spark interest in the modeling community.

The virtual cell [33] which is hosted at the National Resource for Cell Analysis and Modeling is a novel tool that combines computational biology with imaging. The tool allows scientists to model and simulate specific cellular functions from simple molecular motors to complex signaling, in a simple Java environment. At this time, it used together with images in order to model compartments, but this author considers that using HCS data instead of the images themselves could lead to a revolution in the complexity and breadth of cell modeling. Models are always seeking data to both improve the model as well as validate the model. The virtual cell brings together data from physiological models, cellular structures, reactions, fluxes, reactions and so on with spatial data (from images) as well as external data such as pathway analysis and external literature as well as sources such as KEGG [34] (which is a database of the building blocks of biological systems such as, pathways genes and so on). These biological facts are converted into mathematical models, and the simulation engine runs to provide information on time response, steady state data and sensitivities. These data can then be used to drive experiments, refine protocols, and do further modeling. To date, there are approximately 30 published paper using the virtual cell, covering a wide variety of topics from signaling [35] to cell structure dynamics [36] to calcium transport [37].

## SUMMARY – *IN SILICO* BIOLOGY COMES OF AGE

HCS has come a long way in the past 10 years, and informatics and bioinformatics have played a key role, from its early use to perform assays that were intractable without imaging, through phenotype analyses, to today’s genome wide, multi disciplinary studies.

The next steps lie in leveraging this data to build models and perform simulations, as these methods allow the researcher to test many more conditions than are possible in the wet laboratory. It is highly conceivable that a future laboratory could take HCS data from many cell types as source data for pathway models as well as virtual cell models, opening up the tantalizing possibility of modeling gene function, compound mechanism of action and cellular responses in cells and tissues *in silico*, truly achieving the cellome.

## Figures and Tables

**Fig. (1):**
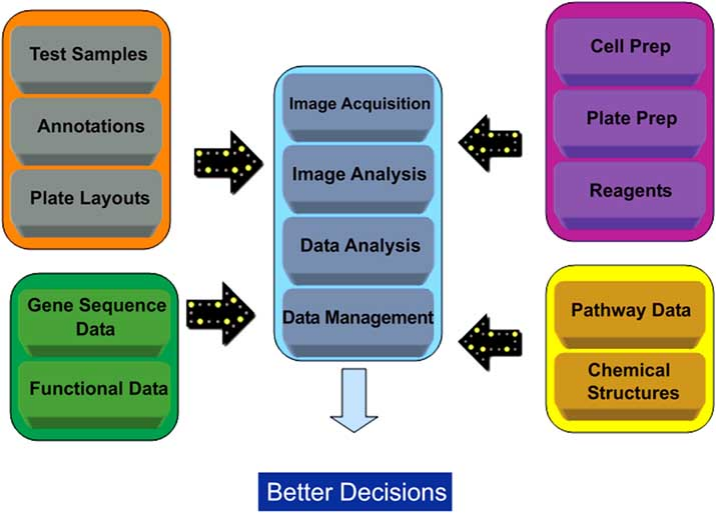
Knowledge generation from HCS data requires that a number of disparate date sources are federated together with HCS data itself in order to make better decisions – HCS data is not an island. Data from HCS experiments is federated together with data about (i) samples, such as sample type, annotations indicating cell type, control type and so on; (ii) data about the cells used, how the plate preparation was performed as well as information about dyes, antibodies and other reagents; (iii) gene sequence data and other functional data (from Medline MESH descriptors for example); (iv) Pathway data and chemical structures also allow cell based SAR, pharmacophore modeling etc., to be layered into HCS knowledge.

**Fig. (4):**
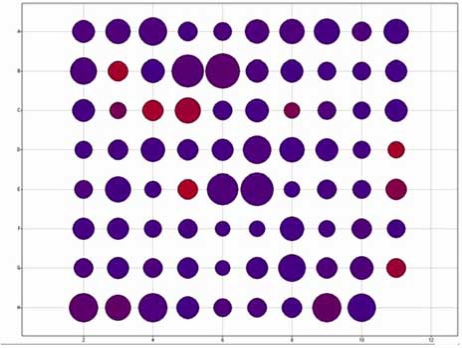
Simple visualization of multiple well level HCS parameters using Spotfire® DecisionSite™. Wells are coloured based on the target parameter (in this case a measure of nuclear intensity) and their diameter is set by the number of targeted cells meeting the assay criteria. Simple visualizations such as these allow ‘at-a-glance’ determinations of multiple parameters, across multiple plates.

**Fig. (5):**
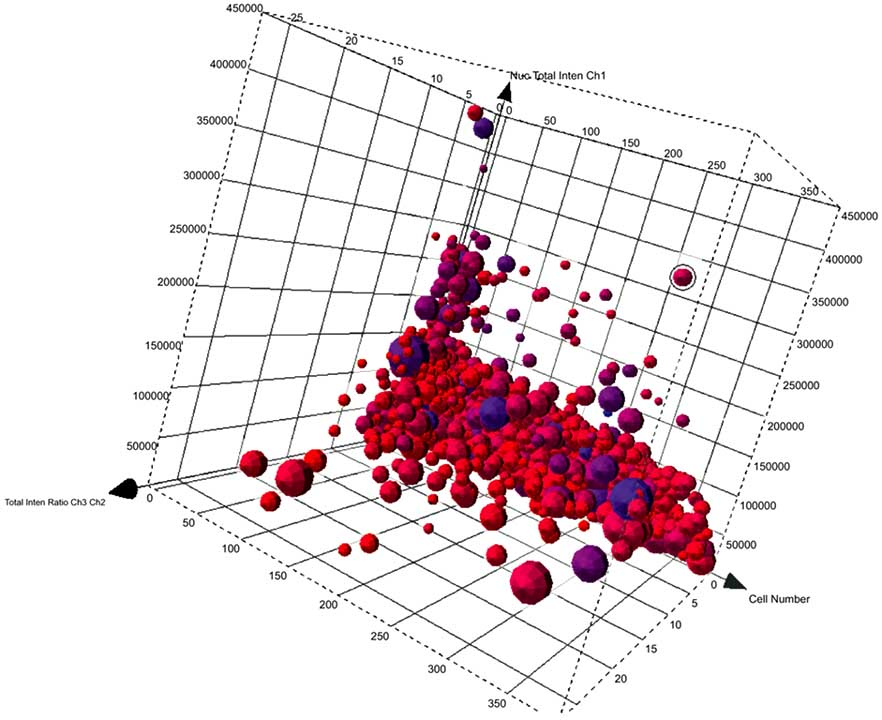
3D visualization of cell cycle parameters in Spotfire® DecisionSite™. 3D plot allows interpretation of 5 parameters, the cell cycle phase (using DNA content), as well as an indicator of which cells might also be stressed based on measurements of shape. Smaller spheres indicate smaller cells. Color is used to provide another indication of shape by measuring cell perimeter to area ratio. In the plot, small, darker shaded spheres represent cells that have rounded up – indicating stress of some kind.

**Fig. (6):**
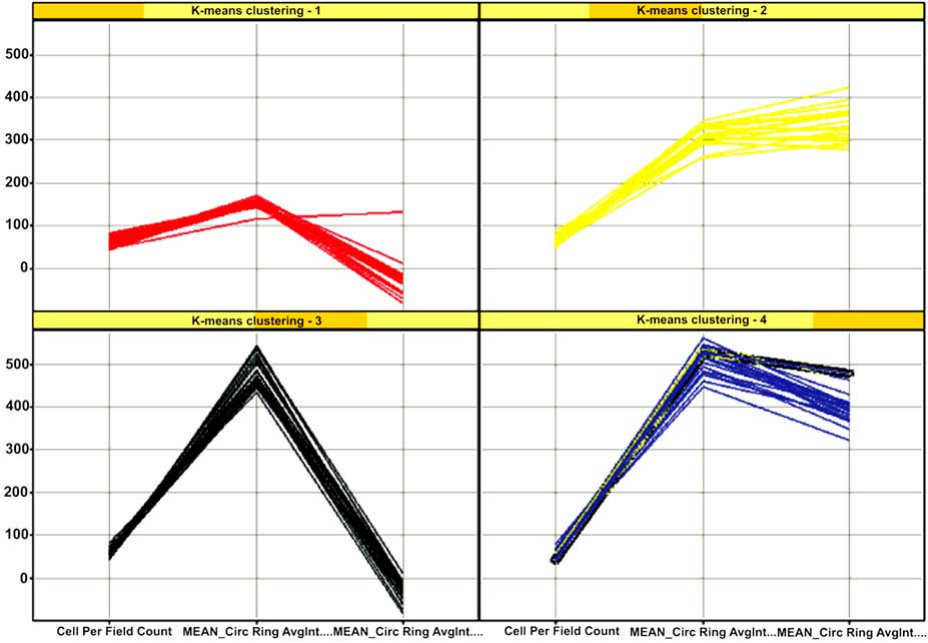
Use of K-nearest neighbor analysis to determine which feature measurements separate stimulated populations of cells from unstimulated populations. K-NN identified 3 measurements able to separate un-stimulated (Red graph, panel **1**) from three types of stimulated populations (Yellow graph panel **2**, Black graph panel **3**, Blue graph panel **4**).

**Table 1.** Useful Statistical and Data Mining Tools for HCS Data. This Table Shows Technique Name and Abbreviation, a Brief Description as Well as Key References where the Technique has been Employed for HCS

Data Mining Technique	Description	References
Kolmogorov-Smirnov (KS) Statistic	The Kolmogorov-Smirnov test (KS-test) is a non-parametric test that tries to determine if two datasets differ significantly. The KS-test has the advantage of making no assumption about the distribution of data, i.e. whether it is normally distributed or not.	[16-18]
Linear Discriminant Analysis (LDA)	Linear Discriminant Analysis (LDA) is a method to discriminate between two or more groups of samples, e.g., controls and samples. The number of groups is not restricted to two, although the discrimination between two groups is the most common approach. Fisher LDA is perhaps the most famous. It has been used in HCS for separating hit and non-hit populations based on multiple cell measurements	[22]
Self Organizing Map (SOM)	A self-organizing map (SOM) is a type of artificial neural network that is trained using unsupervised learning to produce a low-dimensional (typically two dimensional), discretized representation of the input space of the training samples, called a map. It is often used to present multi-dimensional data in a low dimensional (2D) fashion. It has applications in HCS for phenotypic analysis and data discovery as maps may be annotated.	[2]
K-Nearest Neighbor (K-NN)	The k-nearest neighbor algorithm is probably the simplest of all machine learning algorithms and is a method for classifying objects based on closest training examples in the feature space. An object is classified by a majority vote of its neighbors, with the object being assigned to the class most common amongst its k nearest neighbors. K-NN can be used to rapidly create classes based on similarity. HCS data can be rapidly classified into phenotypic classes using this technique.	[22]
Principal Components Analysis	Principal component analysis (PCA) is a mathematical method used to reduce multidimensional data sets to lower dimensions for analysis It is useful as a tool for exploring the classes in data and creating 2D pictures of high dimensional data while allowing the user to discover the principal factors underlying the structure of the data. PCA has been applied to HCS to determine which groups of measurements are related to each other as well as determine key clusters of features in phenotypic data	[15, 21, 42]
Hierarchical Cluster Analysis (HCA)	Hierarchical cluster analysis is a statistical method for finding relatively homogeneous clusters of cases based on measured characteristics. It starts with each case in a separate cluster and then combines the clusters sequentially, reducing the number of clusters at each step until only one cluster is left. It groups like patterns with like patterns and separates those clusters of like patterns from other clusters. It is usually represented as a tree or dendrogram where each step in the clustering process is represented by a node in the tree. It is often used to analyze one set of data, e.g., gene sequence against another (cell measurement) to relate the two together.	[17, 21]
Decision Trees	Decision trees are powerful and popular tools for classification and prediction. The attractiveness of decision trees is due to the fact that, in contrast to neural networks, decision trees represent rules that can be learned from data. Rules can be tested and reviewed by humans rather than the black box approach common in other machine learning approaches (e.g., neural networks). A decision tree is an example of an inductive classifier in the form of a tree structure that uses nodes to represent data and decisions, e.g., “cell is undergoing toxicity if nuclear fragmentation >=50 and membrane permeability <= 100.	[41, 43]
T-test	The t-test assesses whether the means of two groups are *statistically* different from each other. This analysis is appropriate whenever you want to compare the means of two groups, and especially appropriate as the analysis for the determination of control *vs* sample data. Performing a t-test on multiple measurements from an HCS screen can be used to determine (based on t-test score) which feature measurements discriminate between control and sample populations.	[22]
Z prime (Z’)	Z’ is a dimensionless calculation used to assess the quality of a high-throughput assay. It compares the mean value of the maximum signal control to the mean value of the minimum control, and will have a higher value when (a) there is a wide separation band between maximum and minimum controls, and (b) the standard deviations are low. For a good assay, Z’values for each plate should be greater than or equal to 0.5. A perfect assay would have a Z-prime value approaching 1.0. Calculating Z’ values for multiple HCS measurement allows for ranking of measurements that separate minimum signal control from maximum signal controls/samples	[38]
Support Vector Machines (SVM)	SVMs are a set of related supervised learning methods used for classification and regression. A Support Vector Machine (SVM) performs classification by constructing an N-dimensional hyperplane that optimally separates the data into two categories. SVM models are closely related to neural networks. SVMs are particularly tolerant of noisy data sets and build robust classifiers of HCS data.	[22, 25, 39, 40]
